# Leflunomide monotherapy versus combination therapy with conventional synthetic disease-modifying antirheumatic drugs for rheumatoid arthritis: a retrospective study

**DOI:** 10.1038/s41598-020-69309-z

**Published:** 2020-07-23

**Authors:** Daihua Deng, Jun Zhou, Min Li, Siyin Li, Lan Tian, Jinmei Zou, Tingting Wang, Jianhong Wu, Fanxin Zeng, Jing Yang

**Affiliations:** 1 Department of Rheumatology, Mianyang Central Hospital, Mianyang, Sichuan China; 2 Department of Clinical Research Center, Dazhou Central Hospital, No. 56 Nanyuemiao Street, Tongchuan District, Dazhou, Sichuan China; 3 Department of Rheumatology, Dazhou Central Hospital, Dazhou, Sichuan China

**Keywords:** Rheumatic diseases, Drug delivery, Drug safety

## Abstract

Leflunomide (LEF) is a conventional synthetic disease-modifying antirheumatic drugs (csDMARDs) for the treatment of rheumatoid arthritis. However, there are few reports on the comparison of efficacy between LEF alone and combined with other csDMARDs. Here, the efficacy and safety of LEF monotherapy (88) and combination (361) therapy groups were evaluated. After 3 months, there were no significant differences in 28-joint disease activity score (DAS28), health assessment questionnaire (HAQ), erythrocyte sedimentation rate (ESR) and C-reactive protein (CRP) between the monotherapy and combination groups (all *P* > 0.05). According to the European League Against Rheumatism (EULAR) response criteria, it was found that the DAS28 response rates were similar in the two groups (*P* > 0.05). Besides, the two groups presented similar safety profiles. Subgroup analysis found that there was no difference in efficacy among the three combined therapies (LEF + methotrexate (MTX), LEF + hydroxychloroquine (HCQ), and LEF + MTX + HCQ) and LEF monotherapy. Furthermore, when the dose of LEF was less than 40 mg/day, no significant difference in efficacy was observed between low and high doses. Overall, these results indicated that low dose LEF monotherapy was not inferior to the combination therapy.

## Introduction

Rheumatoid arthritis (RA) is a chronic inflammatory autoimmune disease characterized by synovitis, and its primary clinical manifestations is symmetric arthritis affecting multiple joints. Inflammation and joint erosion cause physical function disorder, which severely reduces the quality of life. The global prevalence of RA is 0.5–1%^1,2^, the incidence of RA among females is 2 to 3 times than of males, and the incidence in females shows an increasing trend^3,4^. Although multiple studies have reported that the occurrence of RA is related to environmental, hormonal and genetic factors^5–7^, the specific pathogenesis of RA remains unclear, which is difficult to achieve radical cure. The main goal of treating RA at present is to control and alleviate the disease.

Currently, drugs for treating RA mainly include conventional synthetic disease-modifying antirheumatic drugs (csDMARDs), targeted synthetic DMARDs (tsDMARDs), biological DMARDs (bDMARDs), biosimilar DMARDs (bsDMARDs), and glucocorticoids (GCs)^8^. Economical and effective csDMARDs are the first treatment strategy recommended by the European League Against Rheumatism (EULAR)^8^, including leflunomide (LEF), methotrexate (MTX), hydroxychloroquine (HCQ), sulfasalazine (SSZ), cyclosporine, azathioprine and gold salts. Among them, LEF was approved by the Food and Drug Administration for the treatment of RA in 1998 and plays a role in controlling and relieving the condition of the disease through acting on dihydroorotate dehydrogenase and tyrosine kinase to inhibits pyrimidine synthesis^9,10^. The double-blind randomized trial in RA patients has also found that LEF significantly improves clinical outcomes^11,12^. The recommended dose of LEF is 20 mg/day without loading dose^13^, but studies have shown that the effects of 10 mg/day and 25 mg/day are similar at week 4, and the incidence of adverse events is positively correlated with the dose of LEF^14^. Elevated liver enzymes, diarrhoea, respiratory infections, nausea, skin rash, dyspepsia, headache and alopecia are common adverse caused by LEF^15,16^. Some studies have demonstrated that LEF and MTX have similar effects^15,17,18^. However, the results of a randomized, controlled, single-blind study also suggest that LEF is more effective than MTX^19^. And LEF has a lower incidence of adverse events than MTX^19–21^.

LEF is often used in combination with other drugs in clinic for the treatment of RA^22,23^. But it has been a hot topic of debate as to whether monotherapy and combination therapy has more advantageous therapeutic effects. Previous studies have shown that combination csDMARDs therapy is better than csDMARD monotherapy^22,24–27^, while other studies have reached inconsistent conclusions, and the results have shown that the efficacy of combination csDMARDs therapy is similar to that of the csDMARD monotherapy^28–31^. At present, most studies on csDMARDs mainly focus on the efficacy and toxicity evaluation of MTX monotherapy and/or in combination with other csDMARDs, while few studies report the efficacy of LEF monotherapy or combination.

Therefore, we conducted a retrospective cohort study of patients treated with LEF and objectively evaluated the efficacy and safety of LEF monotherapy and LEF combined with other csDMARDs (MTX and HCQ). And, a stratified analysis was performed to compare the efficacy of different LEF doses therapy.

## Results

### Study population and patient disposition

A total of 449 patients with RA were enrolled in the final analysis, of which 88 were included in the monotherapy group and remaining 361 were included in the combination group. Clinical features of the two groups were similar at baseline (Table 1). In the monotherapy group, 65 patients (73.86%) were female; mean age, 52.15 years; and disease duration was 95.22 months. In the combination group, 294 patients (81.44%) were female; mean age, 50.02 years; and disease duration was 73.48 months. Patients who were administered LEF doses of less than 20 mg accounted for 44.32% in the monotherapy group and 55.40% in the combination group. Furthermore, in the combination group, there were 125 patients who were given LEF + MTX, 88 who were given LEF + HCQ, and 148 who were given LEF + MTX + HCQ.

**Table 1:** Baseline demographics and clinical characteristics of the study cohort.

Characteristic	Monotherapy group (n = 88)	Combination group (n = 361)	*P-*value
**Age, years (mean, SD)**	52.15 (13.35)	50.02 (10.52)	0.140
**Female (n, %)**	65 (73.86%)	294 (81.44%)	0.077
**Male (n, %)**	23 (26.14%)	67 (18.56%)	0.077
**Disease duration, months (mean, range)**	95.22 (4.13–599.83)	73.48 (3.10–450.80)	0.134
**LEF dose/day**			
< 20 mg/day (n, %)	39 (44.32%)	200 (55.40%)	0.074
≥ 20 mg/day (n, %)	49 (55.68%)	161 (44.60%)	0.074
**Therapeutic regimen**			
LEF (n)	88	0	
LEF + MTX (n)	0	125	
LEF + HCQ (n)	0	88	
LEF + MTX + HCQ (n)	0	148	

*LEF* leflunomide, *MTX* methotrexate, *HCQ* hydroxychloroquine.

### Efficacy

**Figure 1:**
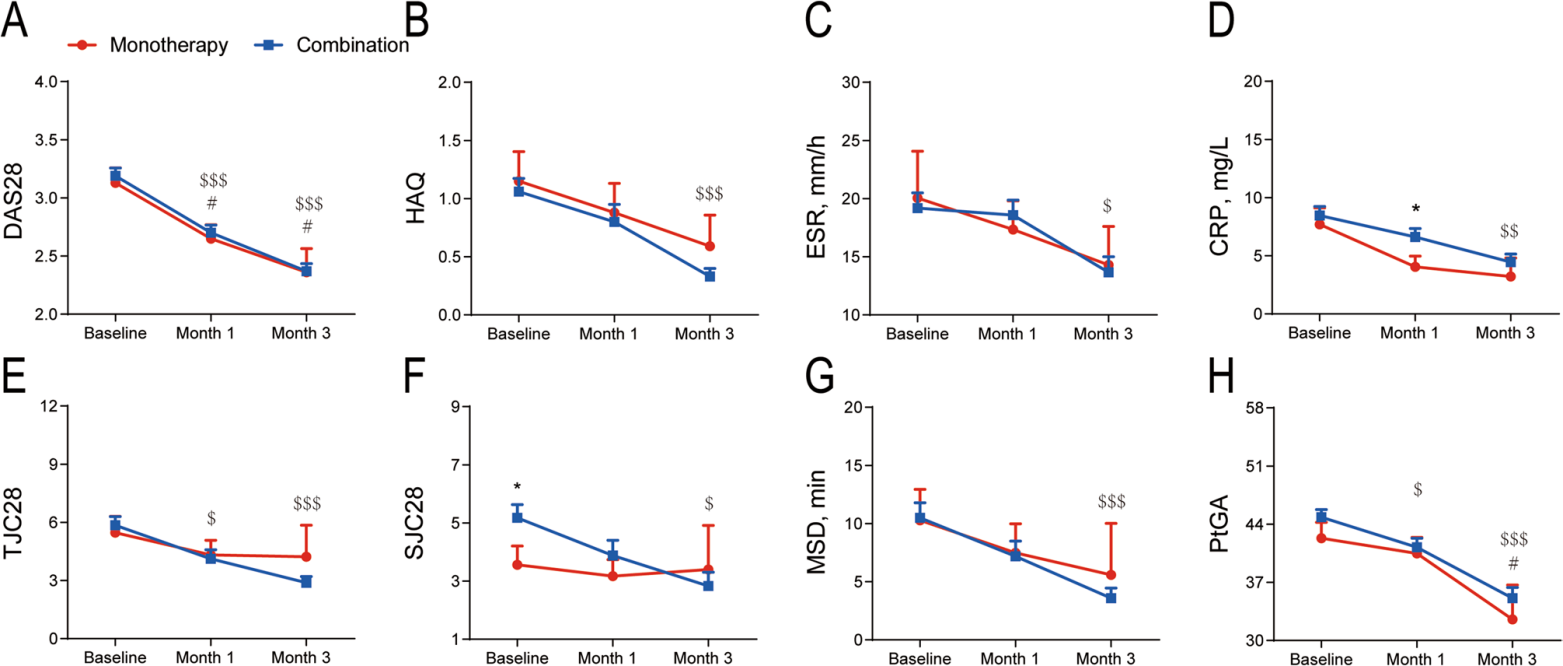
Primary and secondary endpoint results for the monotherapy and combination groups. (**A**) DAS28, (**B**) HAQ, (**C**) ESR, (**D**) CRP, (**E**) TJC28, (**F**) SJC28, (**G**) MSD, (**H**) PtGA. *DAS28* 28-joint disease activity score calculated with C-reactive protein, *HAQ* health assessment questionnaire, *CRP* C-reactive protein, *ESR* erythrocyte sedimentation rate, *TJC28* tender joint count of 28 joints, *SJC28* swollen joint count of 28 joints, *MSD* morning stiffness duration, *PtGA* patient global assessment. Data are shown as means ± SEM. *Indicate significant differences between different groups at the same time point (*P* < 0.05). ^#^Significance as compared with baseline in the monotherapy group (*P* < 0.05). ^$^Significance as compared with baseline in the combination group (^$^*P* < 0.05; ^$$^*P* < 0.01; ^$$$^*P* < 0.001).

After 3 months of follow-up, primary and secondary endpoints in the monotherapy and combination groups were assessed at baseline, month 1 and month 3 (Table 2, Fig. 1). At baseline, the 28-joint disease activity score (DAS28) in the two groups were 3.13 ± 1.21 and 3.19 ± 1.28, respectively, with no significant difference (*P* > 0.05). At month 1, DAS28 was significantly decreased in both groups (monotherapy group: 2.65 ± 0.98, compared with baseline *P* = 0.0216; combination group: 2.70 ± 1.07, compared with baseline *P* < 0.001). At month 3, DAS28 continued to improve (monotherapy group: 2.36 ± 0.96, compared with baseline *P* = 0.0109; combination group: 2.37 ± 0.88, compared with baseline *P* < 0.001). However, there was no statistical difference in DAS28 between the two groups at month 1 and month 3 (*P* > 0.05).

**Table 2:** Clinical outcomes at baseline, month 1 and month 3.

Characteristic	Baseline			Month 1			Month 3		
	Monotherapy group (n = 88)	Combination group (n = 361)	*P*-value	Monotherapy group (n = 68)	Combination group (n = 256)	*P*-value	Monotherapy group (n = 22)	Combination group (n = 187)	*P*-value
**DAS28 core parameter**									
DAS28	3.13 (1.21)	3.19 (1.28)	0.677	2.65 (0.98)	2.70 (1.07)	0.842	2.36 (0.96)	2.37 (0.88)	0.947
TJC28	5.47 (6.32)	5.85 (6.53)	0.405	4.32 (4.43)	4.12 (5.02)	0.830	4.22 (4.89)	2.89 (2.69)	0.668
SJC28	3.56 (4.23)	5.18 (5.64)	0.031*	3.17 (2.79)	3.88 (4.45)	0.754	3.40 (4.79)	2.83 (2.87)	0.942
CRP, mg/L	7.70 (12.60)	8.46 (14.19)	0.678	4.05 (7.17)	6.62 (11.10)	0.021*	3.21 (7.51)	4.45 (9.25)	0.286
**Secondary endpoint**									
PtGA	42.32 (17.25)	44.84 (17.17)	0.265	40.47 (15.36)	41.23 (16.93)	0.746	32.55 (19.36)	35.10 (17.03)	0.515
HAQ	1.15 (2.39)	1.06 (2.16)	0.520	0.88 (2.06)	0.80 (2.41)	0.736	0.59 (1.26)	0.33 (0.95)	0.112
MSD, min	10.28 (21.92)	10.50 (22.16)	0.298	7.48 (19.02)	7.18 (20.15)	0.148	5.59 (18.27)	3.59 (11.40)	0.753
ESR, mm/h	20.06 (35.41)	19.19 (22.88)	0.192	17.35 (18.95)	18.59 (19.93)	0.831	14.29 (15.18)	13.66 (17.43)	0.637

Values are means (SD).

*DAS28* 28-joint disease activity score calculated with C-reactive protein, *TJC28* tender joint count of 28 joints, *SJC28* swollen joint count of 28 joints, *PtGA* patient global assessment, *CRP* C-reactive protein, *HAQ* health assessment questionnaire, *MSD* morning stiffness duration, *ESR* erythrocyte sedimentation rate.

*P < 0.05.

Health assessment questionnaire (HAQ), erythrocyte sedimentation rate (ESR), tender joint count of 28 joints (TJC28), morning stiffness duration (MSD), and patient global assessment (PtGA) had similar results between two groups at baseline, month1 and month 3 (all *P* > 0.05), except for swollen joint count of 28 joints (SJC28) at baseline (*P* = 0.031) and C-reactive protein (CRP) at month 1 (*P* = 0.021) (Table 2, Fig. 1). Compared with baseline, HAQ, ESR, MSD, and PtGA trend toward decreasing in the monotherapy group at month 3, but there was no statistical significance (*P* > 0.05). In the combination group, HAQ, ESR, MSD, and PtGA decreased significantly at month 3 compared with baseline (*P* < 0.05).

**Figure 2:**
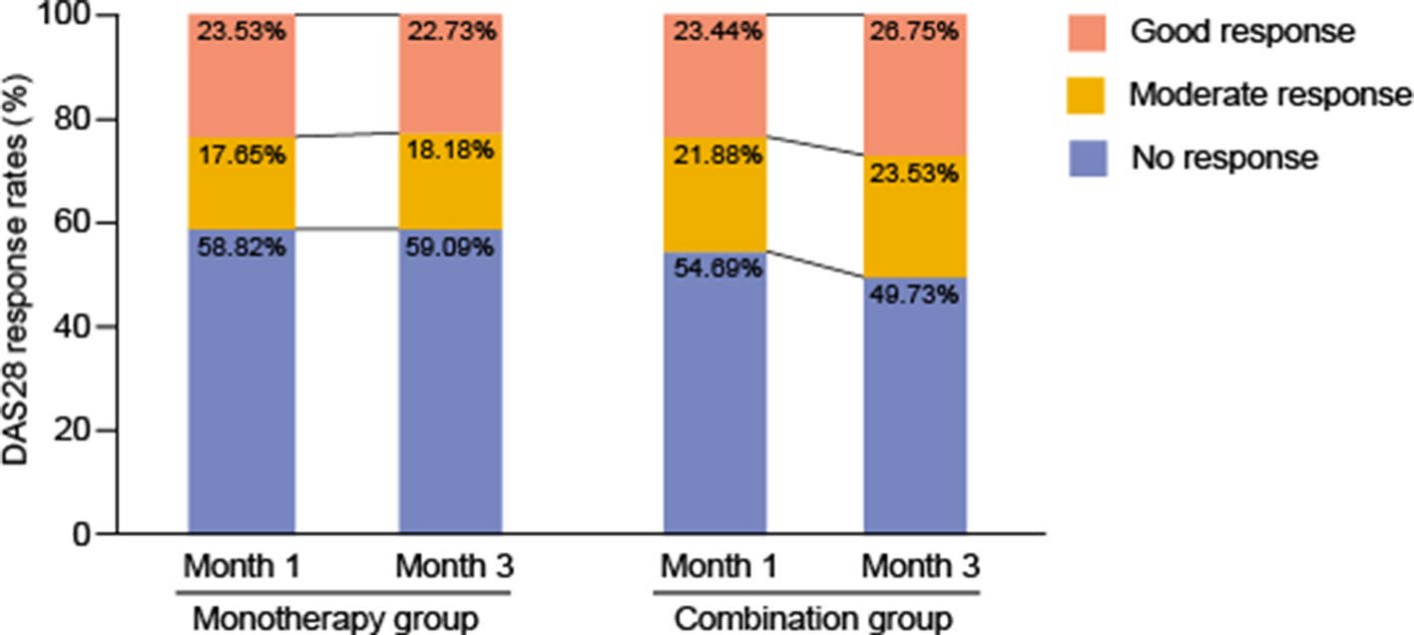
DAS28 response rates in the monotherapy and combination groups.

DAS28 response rate was observed in the monotherapy group and the combination group at month 1 and month 3 (Fig. 2). At month 1, 23.53% of patients in the monotherapy group had a good response, 17.65% had a moderate response and 58.82% had no response according to EULAR criteria. The combination group showed similar results with 23.44%, 21.88% and 54.69% having a good response, moderate response and no response, respectively. At month 3, the proportion of good response in the monotherapy group (23.44%) was lower than that in the combination group (26.75%). Still, the composition ratios of no response, moderate response and good response were not significantly different between the two groups (*P* > 0.05). Taken together, these results confirmed that there was no significant difference in efficacy between the monotherapy group and the combination therapy group at month 1 and month 3.

### Safety

The hematological examination results before and after administration of monotherapy and combination therapy are shown in Table 3. Regardless of whether patients were in the monotherapy group or the combination group, the four blood routine indicators (hemoglobin (HGB), red blood cell (RBC), mean corpuscular volume (MCV), and white blood cell (WBC) showed similar results before and after the treatment. There were also no significant differences in liver function (albumin (ALB), aspartate transaminase (AST) and alanine transaminase (ALT)) or kidney function indicators (glomerular filtration rate (GFR) and serum creatinine (Scr)). Multiple hematological examination results before and after treatment revealed that the safety was similar in the monotherapy and combination groups.

**Table 3:** Changes of hematological examination before and after medication.

Characteristic	Monotherapy group			Combination group		
	Premedication (n = 43)	Post-medication (n = 18)	Δ	Premedication (n = 151)	Post-medication (n = 106)	**Δ**
**Blood routine**						
HGB, g/L	128.83 (15.58)	131.92 (13.07)	3.09	123.91 (15.96)	123.83 (19.70)	− 0.08
RBC, 10^12^/L	4.24 (0.39)	4.33 (0.38)	0.09	4.22 (0.61)	4.26 (0.58)	0.04
MCV, fL	92.71 (4.68)	94.26 (6.83)	1.55	91.45 (7.25)	91.66 (8.21)	0.21
WBC, 10^9^/L	6.07 (1.72)	5.63 (1.32)	− 0.44	6.21 (2.06)	5.46 (1.85)	− 0.75
**Liver results**						
ALB, g/L	43.92 (5.10)	45.04 (3.86)	1.12	43.15 (4.07)	43.72 (4.23)	0.57
AST, U/L	23.10 (9.87)	23.47 (7.73)	0.37	24.17 (10.80)	27.31 (13.64)	3.14
ALT, U/L	19.97 (7.34)	27.89 (17.95)	7.92	22.83 (12.21)	26.85 (14.99)	4.02
**Kidney results**						
GFR, mL/min	84.70 (23.77)	94.83 (13.53)	10.13	89.60 (28.84)	86.85 (24.14)	− 2.75
Scr, μmol/L	61.98 (11.86)	52.09 (10.87)	− 9.89	53.11 (10.89)	58.21 (19.39)	5.10

Values are means (SD).

*HGB* hemoglobin, *RBC* red blood cell, *MCV* mean corpuscular volume, *WBC* white blood cell, *ALB* albumin, *AST* aspartate transaminase, *ALT* alanine transaminase, *GFR* glomerular filtration rate, *Scr* serum creatinine, *Δ* the change before and after medication.

### Subgroup analysis

The combination group included three treatment regimens, and we compared the efficacy of the four medication regimens in the treatment of RA (Supplementary Table S1, Supplementary Fig. S1). At baseline, the DAS28 were 3.13 ± 1.21 (LEF), 3.37 ± 1.29 (LEF + MTX), 2.89 ± 1.21 (LEF + HCQ) and 3.22 ± 1.27 (LEF + MTX + HCQ), respectively, with no significant difference (*P* > 0.05). DAS28, HAQ, ESR, CRP, TJC28, SJC28 and PtGA in the four groups had similar results at month 1 and month 3 (all *P* > 0.05), except for MSD at baseline. Thus, there was no significant difference in efficacy of the four regimens at month 1 and month 3.

We further analyzed the efficacy of LEF in patients who received < 20 mg/day (low dose) or ≥ 20 mg/day (high dose) of LEF in the monotherapy group and in the combination group (Supplementary Fig. S2). DAS28, HAQ, ESR, CRP, TJC28, MSD and PtGA were remarkably similar among the four groups at baseline, month 1 and month 3. Except that, SJC28 was significantly lower in the high dose monotherapy group at baseline. In summary, there were no significant differences in the primary and secondary endpoints among the four groups at baseline, month 1 and month 3 (*P* > 0.05).

## Discussion

In the retrospective cohort study, we mainly compared the efficacy between RA patients with LEF monotherapy and LEF combination therapy with csDMARDs. Our study reported that there were no significant differences in the primary endpoint DAS28 and multiple secondary endpoints between the monotherapy group and the combination group after 3 months of treatment. Both groups also have similar safety. In the subgroup analysis of treatment regimen, clinical outcomes in patients treated with LEF, LEF + MTX, LEF + HCQ, and LEF + MTX + HCQ was similar. When the dose of LEF was in the high dose or low dose levels, the dose of LEF had no significant effect on the efficacy of the treatment regimen, either alone or combined with other csDMARDs.

LEF, typical csDMARDs, effectively inhibits the activated immune response and has good efficacy and safety in the treatment of RA^9,32^. Compared with placebo, LEF significantly improved clinical outcomes in RA patients at month 1 and month 3^11^. SSZ was similar to LEF in terms of CRP, MSD and HAQ after 6 months^11,12^. In our study, DAS28 and PtGA were significantly reduced in the monotherapy group (LEF) at month 3 compared to baseline, which suggested that LEF was an effective drug for RA treatment. In the clinical therapy of RA, the recommended dose for LEF is 20 mg/day^13^. However, Mladenovic et al. came to a conclusion that there was no significant difference in efficacy between 10 mg/day and 20 mg/day LEF after 4 weeks treatment. When the daily dose of LEF was 5 mg, the effect was similar to placebo^14^. Our results showed that there was no significant difference in efficacy between low dose LEF (< 20 mg/day) and high dose LEF (≥ 20 mg/day) after 1 month and 3 months therapy. The low dose was mainly 10 mg/day, while the high dose was mainly 20 mg/day. LEF dose was positively correlated with liver enzyme elevation and adverse events^14,33^, so low dose LEF was worth considering within the same efficacy.

In treatment strategies for RA, drugs are often used in combination to prevent disease progression^8^. However, a study in RA patients with step-up therapy strategy demonstrated that LEF monotherapy and combination therapy has been similar efficacy, that was, there was no significant difference in DAS28 response rate and American College of Rheumatology (ACR) 20 score between LEF + SSZ and SSZ + placebo after 6 months of treatment^31^. Our study was a parallel strategy in which the combination group received one or more csDMARDs simultaneously. There was no significant difference in efficacy of the combination group at month 1 and month 3 compared to the LEF monotherapy group. Both studies with different study designs substantiated that LEF monotherapy was not inferior to csDMARDs combined therapy. However, LEF combined with drugs that have different mechanisms of action may improve therapy efficacy, for instance integrative LEF and rituximab is significantly more effective than rituximab alone^34^. Therefore, the combination of LEF with drugs with different modes of action seems to increase the benefits more than with csDMARDs.

As a chronic disease, RA is generally with long treatment period and complicated medication regimen. It was inevitable in our study that patients took other drugs before the assessment. However, the disease activity of the two therapy groups at baseline was at the same level, and there was no significant influence on the efficacy evaluation of LEF in this study. Of note, we excluded patients who took GCs to avoid the influence of GCs on the evaluation of efficacy. Although some studies have found that GCs included in treatment do not have a significant impact on the efficacy of MTX + GCs or MTX + GCs + other csDMARDs (LEF or SSZ or SSZ + HCQ)^29,30^, other studies included GCs treatment regimen have different opinions, which have shown that combination therapy was better than monotherapy^25,35^. Different conclusions might be partly caused by differences in study designs^36^, treatment regimens, observation time and evaluation indicators. Therefore, our study reduced confounding factors to some extent. Besides, we counted several indicators, such as DAS28, HAQ, ESR, CRP, TJC28, SJC28 and DAS28 response rate in our study. This facilitates comparisons between studies.

The combination of multiple drugs usually increases drug toxicity^20^, but our study found that there was no significant change in blood routine, liver and kidney function in the monotherapy group or the combination group before and after medication. Therefore, the safety of the two groups was similar. Analogous conclusion was reached in the study of Kremer et al. where the incidence of adverse events was similar between the two groups (LEF + MTX and MTX + placebo)^22^.

Treatment-driven data inevitably had some potential limitations. First, some patients were lost to follow-up in this retrospective study. At month 3, there were only 22 patients in the monotherapy group, which might result in our conclusion less representativeness and universality. Second, a total of 3 months from baseline to the end of observation was relatively short and evaluation indicators was relatively less. To address these limitations, we should collect complete data from multiple hospitals to increase the sample size, increase follow-up time to help us better understand the dynamic changes of the condition and add other evaluation indicators to more objectively evaluate the efficacy of the drug, such as the simplified disease activity index and clinical disease activity index. Third, the combination group contained three treatment regimens, which resulted in the sample imbalance between the combination group and the monotherapy group, and might have a certain impact on the statistical power. However, in the subgroup analysis of treatment regimens, the combination group was divided into three subgroups, with a relatively balanced number of patients in each group, which reduced the impact of sample inequality on the results. And, the result of subgroup analysis was consistent with the main conclusion of LEF monotherapy versus combination therapy comparison, that was, the efficacy of LEF monotherapy was not inferior to LEF combination therapy.

In conclusion, the efficacy of LEF monotherapy and LEF combined with csDMARDs was similar. However, LEF monotherapy was a more economical treatment regimen than combination therapy. At the same time, the low dose of LEF (10 mg/day) was also worth considering.

## Methods

### Patients

Data on patients with RA were collected at Mianyang Central Hospital in southwest China from January 2015 to June 2019. The study was based on clinical data and independently reviewed and approved by the local ethics committee (Ethics Committee of Dazhou Central Hospital, approval number: IRB00000003-19003). As this study was not risky for the subjects, the Ethics Committee waived the need for patients to sign informed consent. All methods were carried out in accordance with relevant guidelines and regulations.

**Figure 3:**
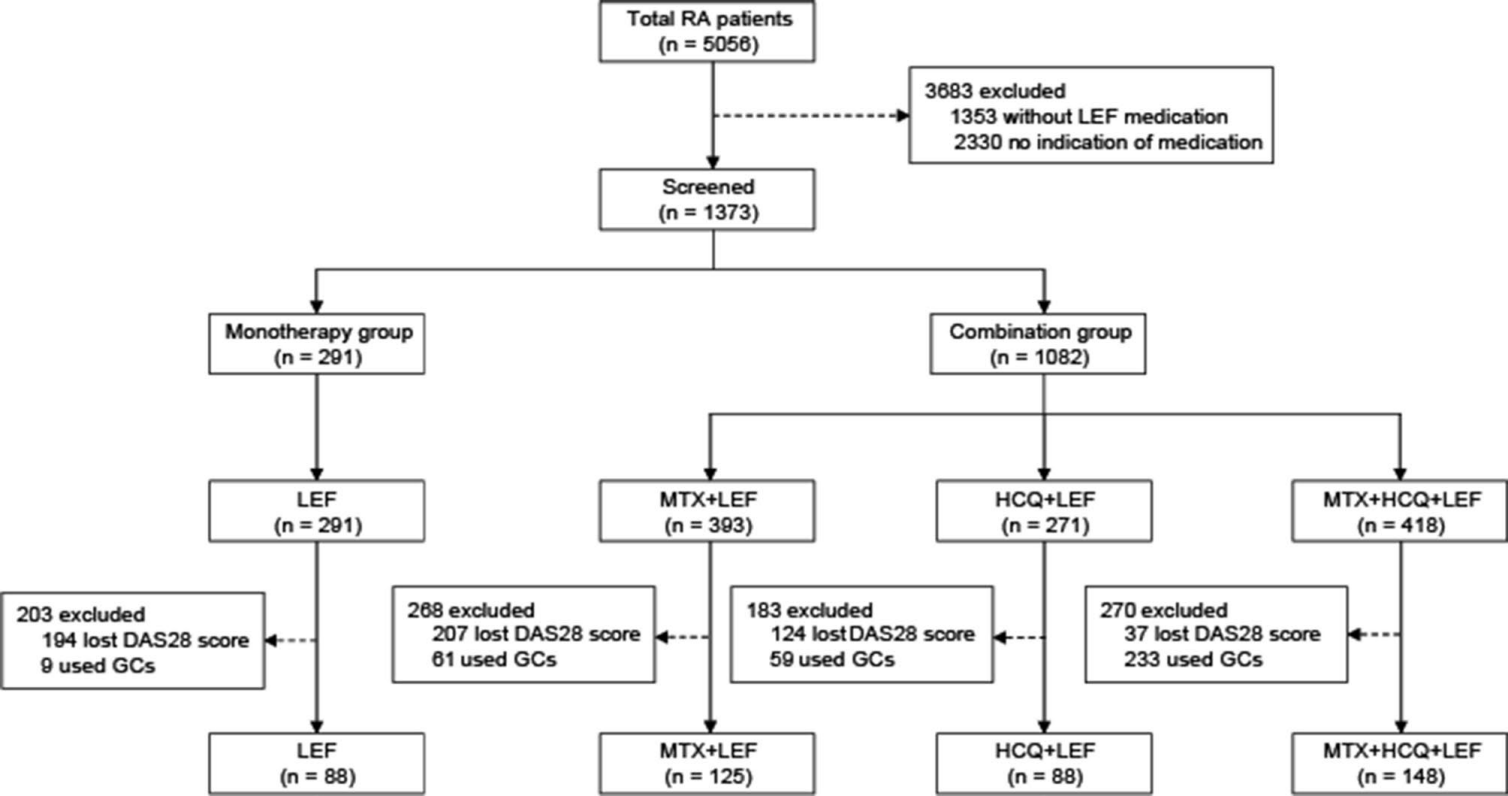
Selection of the study population. *RA* rheumatoid arthritis, *DAS28* 28-joint disease activity score calculated with C-reactive protein, *LEF* leflunomide, *MTX* methotrexate, *HCQ* hydroxychloroquine, *GCs* glucocorticoids.

Patients over 18 years of age and diagnosed with RA based on the ACR 1987 revised criteria were retrospectively enrolled in the study^37^. Exclusion criteria mainly included the following: (1) patients without medication information; (2) Patients did not receive LEF; (3) patients had taken GCs; (4) the time of medication did not match the assessment time of the primary endpoint; (5) missing DAS28 score calculated with CRP. Patients who had complications such as diabetes, hypertension, and osteoporosis were not excluded. Patients taking non-steroidal anti-inflammatory drugs such as ibuprofen, meloxicam or diclofenac were not excluded. The detailed exclusion process is shown in Fig. 3.

### Study design

This retrospective study was followed for 3 months after treatment. The primary and secondary endpoints were assessed at baseline, month 1 and month 3, respectively. According to the medication situation of patients, they were mainly divided into two groups, namely monotherapy group and combination group. The monotherapy group received only LEF, and the combination group received two or three csDMARDs (LEF, MTX or HCQ), which must contain LEF. In the study cohort, the dose of MTX administered was 10.44 ± 2.93 mg/week (median 10 mg/week). The dose of LEF administered was 14.47 ± 5.33 mg/day (median 10 mg/day). The dose of HCQ administered was 374.58 ± 80.62 mg/day (median 400 mg/day).

### Subgroups

To further evaluate the efficacy between specific treatment regimens, we performed a subgroup analysis of the combination group. There were three drug regimens included in the subgroup. The first therapeutic regimen was LEF combined with MTX; the second therapeutic regimen was LEF combined with HCQ; the third therapeutic regimen was LEF combined with MTX and HCQ.

In addition, a subgroup analysis was performed based on the dose of LEF, low dose group (< 20 mg/day) and high dose group (≥ 20 mg/day). The median doses of LEF was 10 mg/day (5–15 mg/day) in the low dose group and 20 mg/day (20–40 mg/day) in the high dose group.

### Study endpoints

The primary endpoint was DAS28 at month 3. The secondary endpoints were HAQ, ESR, CRP, TJC28, SJC28, MSD, PtGA and DAS28 response rate. We used EULAR response criteria to calculate the DAS28 response rate after treatment^38,39^. Safety was evaluated based on multiple hematological examinations. The hematological examination included HGB, WBC, MCV, WBC, ALB, AST, ALT, GFR and Scr.

### Statistical analysis

Statistical analysis was performed using IBM SPSS Statistics 20.0. GraphPad Prism 8 was used for drawing. Overall differences between groups were tested using the Student’s *t* test or two-way analysis of variance (ANOVA) with Tukey's multiple comparisons test depending on distribution of the variables. Non-normal distribution and continuous variables were tested using the Mann–Whitney *U* test. Categorical variables were tested with Chi-square test. A two-sided *P*-value < 0.05 was considered statistically significant.

## Supplementary information

Supplementary Figures

Supplementary Table
