# Supplementary material for: Leflunomide monotherapy versus combination therapy with conventional synthetic disease-modifying antirheumatic drugs for rheumatoid arthritis: a retrospective study
Source: Sci Rep. 2020 Jul 23;10:12339. doi: 10.1038/s41598-020-69309-z (PMC7378063; doi:10.1038/s41598-020-69309-z)
Supplement: Supplementary file 1 — Supplementary Figures [file 41598_2020_69309_MOESM1_ESM.docx]

**Title:**

Leflunomide monotherapy versus combination therapy with conventional synthetic disease-modifying antirheumatic drugs for rheumatoid arthritis: a retrospective study

**Authors:**

Daihua Deng^1,*^, Jun Zhou^2,*^, Min Li^1^, Siyin Li^1^, Lan Tian^1^, Jinmei Zou^1^, Tingting Wang^3^, Jianhong Wu^3^, Fanxin Zeng^2,#^, Jing Yang^1,#^

**Affiliation:**

^1^Department of Rheumatology, Mianyang Central Hospital, Mianyang, Sichuan, China.

^2^Department of Clinical Research Center, Dazhou Central Hospital, Dazhou, Sichuan, China.

^3^Department of Rheumatology, Dazhou Central Hospital, Dazhou, Sichuan, China

*These authors contribute equally.

**#Corresponding Author:**

Jing Yang, M.D.

Department of Rheumatology, Mianyang Central Hospital, Mianyang, Sichuan, China.

Email: yangjing6525@163.com

Fanxin Zeng, Ph.D.

No.56 Nanyuemiao Street, Tongchuan District, Dazhou, Sichuan province, P. R. China

E-Mail: zengfx@pku.edu.cn

Tel: 086-0818-2381051


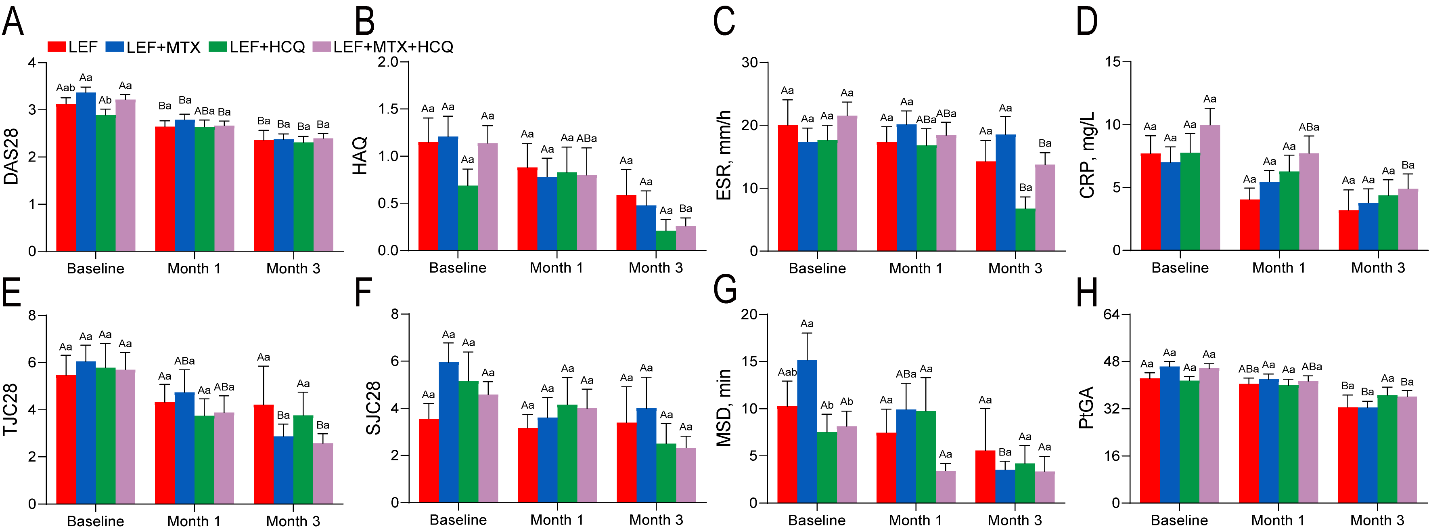


**Supplementary Figure S1.** Primary and secondary endpoint results for the four treatment regimens. (A) DAS28, (B) HAQ, (C) ESR, (D) CRP, (E) TJC28, (F) SJC28, (G) MSD, (H) PtGA. DAS28, 28-joint disease activity score calculated with C-reactive protein; HAQ, health assessment questionnaire; CRP, C-reactive protein; ESR, erythrocyte sedimentation rate; TJC28, tender joint count of 28 joints; SJC28, swollen joint count of 28 joints; MSD, Morning stiffness duration; PtGA, patient global assessment. Data are shown as means ± SEM. Different lowercase letters indicate significant differences between different groups at the same time (*P* < 0.05), different capital letters indicate significant differences between the same group at different times (*P* < 0.05).


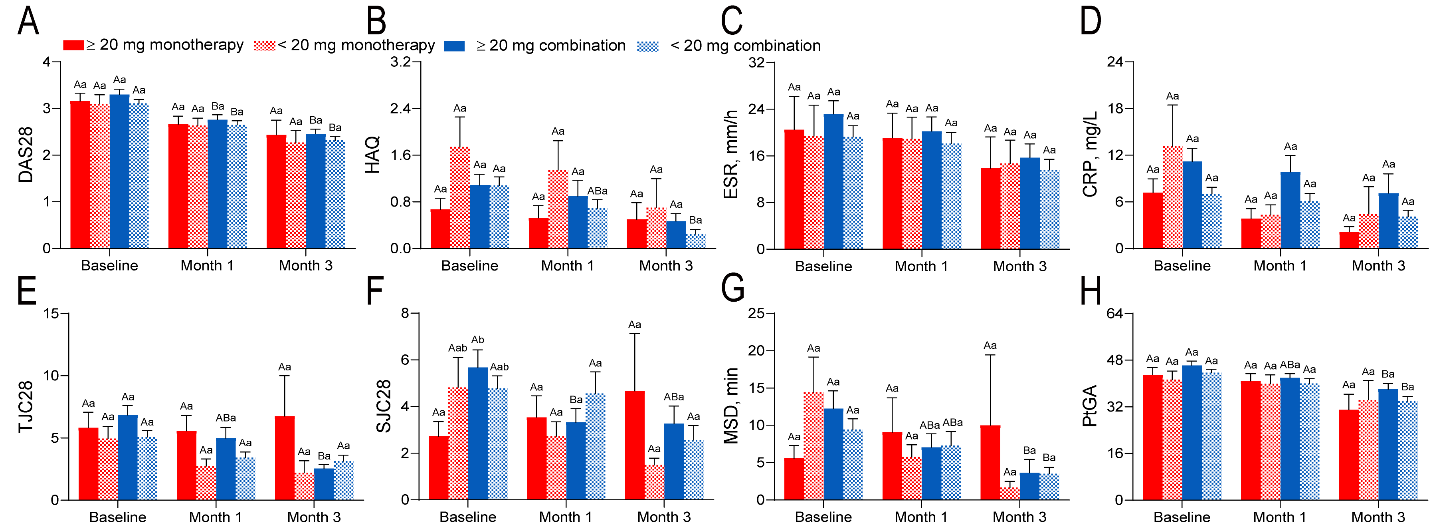


**Supplementary Figure S2.** Effect of the dose of LEF on the efficacy of the monotherapy group and the combination group. (A) DAS28, (B) HAQ, (C) ESR, (D) CRP, (E) TJC28, (F) SJC28, (G) MSD, (H) PtGA. DAS28, 28-joint disease activity score based on C-reactive protein; HAQ, health assessment questionnaire; CRP, C-reactive protein; ESR, erythrocyte sedimentation rate; TJC28, tender joint count of 28 joints; SJC28, swollen joint count of 28 joints; MSD, Morning stiffness duration; PtGA, patient global assessment. Data are shown as means ± SEM. Different lowercase letters indicate significant differences between different groups at the same time (*P* < 0.05), different capital letters indicate significant differences between the same group at different times (*P* < 0.05).
