# Supplementary material for: Leflunomide monotherapy versus combination therapy with conventional synthetic disease-modifying antirheumatic drugs for rheumatoid arthritis: a retrospective study
Source: Sci Rep. 2020 Jul 23;10:12339. doi: 10.1038/s41598-020-69309-z (PMC7378063; doi:10.1038/s41598-020-69309-z)
Supplement: Supplementary file 2 — Supplementary Table [file 41598_2020_69309_MOESM2_ESM.docx]

**Title:**

Leflunomide monotherapy versus combination therapy with conventional synthetic disease-modifying antirheumatic drugs for rheumatoid arthritis: a retrospective study

**Authors:**

Daihua Deng^1,*^, Jun Zhou^2,*^, Min Li^1^, Siyin Li^1^, Lan Tian^1^, Jinmei Zou^1^, Tingting Wang^3^, Jianhong Wu^3^, Fanxin Zeng^2,#^, Jing Yang^1,#^

**Affiliation:**

^1^Department of Rheumatology, Mianyang Central Hospital, Mianyang, Sichuan, China.

^2^Department of Clinical Research Center, Dazhou Central Hospital, Dazhou, Sichuan, China.

^3^Department of Rheumatology, Dazhou Central Hospital, Dazhou, Sichuan, China

*These authors contribute equally.

**#Corresponding Author:**

Jing Yang, M.D.

Department of Rheumatology, Mianyang Central Hospital, Mianyang, Sichuan, China.

Email: yangjing6525@163.com

Fanxin Zeng, Ph.D.

No.56 Nanyuemiao Street, Tongchuan District, Dazhou, Sichuan province, P. R. China

E-Mail: zengfx@pku.edu.cn

Tel: 086-0818-2381051

**Supplementary Table S1.** Primary and secondary endpoint results for the four therapeutic regimens. Values are mean (SD). DAS28, 28-joint disease activity score calculated with C-reactive protein; TJC28, tender joint count of 28 joints; SJC28, swollen joint count of 28 joints; CRP, C-reactive protein; PtGA, patient global assessment; HAQ, health assessment questionnaire; MSD, Morning stiffness duration; ESR, erythrocyte sedimentation rate.

|  | **Baseline** | | | | **Month 1** | | | | **Month 3** | | | |
| --- | --- | --- | --- | --- | --- | --- | --- | --- | --- | --- | --- | --- |
| **Characteristic** | **LEF (n = 88)** | **LEF+MTX (n = 125)** | **LEF+HCQ (n = 88)** | **LEF+MTX+HCQ (n = 148)** | **LEF (n = 68)** | **LEF+MTX (n = 88)** | **LEF+HCQ (n = 63)** | **LEF+MTX+HCQ (n = 105)** | **LEF (n = 22)** | **LEF+MTX (n = 64)** | **LEF+HCQ (n = 39)** | **LEF+MTX+HCQ (n = 84)** |
| DAS28 core parameter |  |  |  |  |  |  |  |  |  |  |  |  |
| DAS28 | 3.13 (1.21) | 3.37 (1.29) | 2.89 (1.21) | 3.22 (1.27) | 2.65 (0.98) | 2.79 (1.11) | 2.64 (1.15) | 2.67 0.99) | 2.36 (0.96) | 2.38 (0.89) | 2.31 (0.81) | 2.40 (0.90) |
| TJC28 | 5.47 (6.32) | 6.05 (6.10) | 5.79 (6.70) | 5.70 (6.87) | 4.32 (4.43) | 4.74 (5.90) | 3.75 (4.03) | 3.88 (4.92) | 4.22 (4.89) | 2.88 (2.57) | 3.77 (3.54) | 2.58 (2.43) |
| SJC28 | 3.56 (4.23) | 5.96 (6.06) | 5.16 (6.97) | 4.59 (4.52) | 3.17 (2.79) | 3.61 (4.52) | 4.16 (5.01) | 4.00 (4.07) | 3.40 (4.79) | 4.00 (4.16) | 2.50 (2.45) | 2.33 (2.06) |
| CRP, mg/L | 7.70 (12.60) | 7.01 (12.48) | 7.78 (14.03) | 9.98 (15.41) | 4.05 (7.17) | 5.47 (7.77) | 6.28 (10.23) | 7.73 (13.56) | 3.21 (7.51) | 3.79 (7.97) | 4.38 (7.71) | 4.90 (10.65) |
| Secondary outcome |  |  |  |  |  |  |  |  |  |  |  |  |
| PtGA | 42.32 (17.25) | 46.36 (17.19) | 41.55 (13.85) | 45.71 (18.76) | 40.47 (15.36) | 42.05 (15.71) | 39.97 (16.51) | 41.39 (18.18) | 32.55 (19.36) | 32.46 (16.18) | 36.69 (16.42) | 36.17 (17.86) |
| HAQ | 1.15 (2.39) | 1.21 (2.37) | 0.69 (1.63) | 1.14 (2.24) | 0.88 (2.06) | 0.78 (1.84) | 0.83 (2.11) | 0.80 (2.95) | 0.59 (1.26) | 0.48 (1.22) | 0.21 (0.73) | 0.26 (0.79) |
| MSD, min | 10.28 (21.92) | 15.18 (28.96) | 7.54 (15.37) | 8.16 (17.75) | 7.48 (19.02) | 9.94 (25.04) | 9.75 (25.93) | 3.43 (7.71) | 5.59 (18.27) | 3.54 (7.03) | 4.21 (11.07) | 3.36 (14.11) |
| ESR, mm/h | 20.06 (35.41) | 17.41 (21.88) | 17.67 (21.00) | 21.53 (24.68) | 17.35 (18.95) | 20.17 (18.97) | 16.84 (20.55) | 18.44 (20.38) | 14.29 (15.18) | 18.55 (20.57) | 6.77 (11.46) | 13.78 (16.60) |
